# Geospatial based model for malaria risk prediction in Kilombero valley, South-eastern, Tanzania

**DOI:** 10.1371/journal.pone.0293201

**Published:** 2023-10-24

**Authors:** Stephen P. Mwangungulu, Deus Dorothea, Zakaria R. Ngereja, Emmanuel W. Kaindoa

**Affiliations:** 1 Department of Geospatial Science and Technology, Ardhi University, Dar es Salaam, United Republic of Tanzania; 2 Environmental Health and Ecological Sciences Department, Ifakara Health Institute, Ifakara, United Republic of Tanzania; 3 The Nelson Mandela, African Institution of Science and Technology, School of Life Sciences and Bio Engineering, Tengeru, Arusha, United Republic of Tanzania; 4 Wits Research Institute for Malaria, School of Pathology, Faculty of Health Sciences, University of the Witwatersrand and the Centre for Emerging Zoonotic and Parasitic Diseases, National Institute for Communicable Diseases, Johannesburg, South Africa; University of Glasgow College of Medical Veterinary and Life Sciences, UNITED KINGDOM

## Abstract

**Background:**

Malaria continues to pose a major public health challenge in tropical regions. Despite significant efforts to control malaria in Tanzania, there are still residual transmission cases. Unfortunately, little is known about where these residual malaria transmission cases occur and how they spread. In Tanzania for example, the transmission is heterogeneously distributed. In order to effectively control and prevent the spread of malaria, it is essential to understand the spatial distribution and transmission patterns of the disease. This study seeks to predict areas that are at high risk of malaria transmission so that intervention measures can be developed to accelerate malaria elimination efforts.

**Methods:**

This study employs a geospatial based model to predict and map out malaria risk area in Kilombero Valley. Environmental factors related to malaria transmission were considered and assigned valuable weights in the Analytic Hierarchy Process (AHP), an online system using a pairwise comparison technique. The malaria hazard map was generated by a weighted overlay of the altitude, slope, curvature, aspect, rainfall distribution, and distance to streams in Geographic Information Systems (GIS). Finally, the risk map was created by overlaying components of malaria risk including hazards, elements at risk, and vulnerability.

**Results:**

The study demonstrates that the majority of the study area falls under moderate risk level (61%), followed by the low risk level (31%), while the high malaria risk area covers a small area, which occupies only 8% of the total area.

**Conclusion:**

The findings of this study are crucial for developing spatially targeted interventions against malaria transmission in residual transmission settings. Predicted areas prone to malaria risk provide information that will inform decision-makers and policymakers for proper planning, monitoring, and deployment of interventions.

## Background

Vector control tools such as Long Lasting Insecticide-treated Nets (LLINs) and Indoor Residual Sprays (IRS) have significantly contributed to the reduction of malaria transmission [1, 2]. However, after more than a decade of success in controlling malaria transmission, the progress has stalled, and no major gains were made globally in reducing malaria cases in the period between 2015 and 2021 [3] due to several challenges, including emerging insecticide resistance, changing in mosquitoes behavior, human behaviors and Covid-19 disruptions [3].

Tanzania is an example of a country that has made remarkable efforts to tackle malaria. Evidence shows that malaria prevalence among children was significantly reduced by more than 50% between 2000 and 2017 [1]. According to the Tanzania Malaria Indicator Survey (TMIS), prevalence was estimated to be 18.0% in children under five in 2008 [1, 4], but was reduced to 7.3% in 2017 [4]. This reduction is mainly attributed to the scale up of long-lasting insecticide-treated bed nets (LLINs) [1]. These nets have been supplied through different campaigns, such as the Tanzania National Voucher Scheme (TNVS) in 2004, mass distribution campaigns, Universal Coverage Campaigns (UCC) and School Net Program (SNP) [5] and other control measures, such as the application of indoor residual-sprays (IRS) [6]. Furthermore, malaria reduction is attributed to advanced diagnostic tools and access to health care [7], proper medication, and overall economic growth and urbanization. Despite the significant efforts made, the persistence of low-level malaria transmission, also known as residual transmission in the community remains a major challenge [8]. While Tanzania is aiming to eliminate malaria by 2030 [9], new efforts are necessary to identify places that sustain high malaria transmission. Furthermore, there is a need for cost-effective use of limited resources, targeting important hotspots even at fine-scale levels, so as to accelerate malaria elimination.

The Kilombero valley in South-eastern Tanzania consistently experiences meso-endemic malaria and maintains high mosquito densities throughout the year, with the peak occurring between January and May. Historical evidence suggests that individuals in this area were subjected to over 1,000 infectious mosquito bites per person per year. However, current evidence indicates a significant decline in malaria transmission rates. Presently, the primary malaria vectors in this area are *Anopheles arabiensis* and *Anopheles funestus*, as the use of insecticidal nets has nearly eradicated *Anopheles gambiae* s.s [10, 11]. In addition to these species, there are various other *Anopheles* mosquitoes, which are known to be secondary malaria vectors, as well as culicine species, particularly *Mansonia*, *Aedes*, and *Culex* mosquitoes. The major malaria intervention is LLINs. Interestingly, the mosquito vectors in this region are most active outdoors during times when people are also outdoors engaging in various activities at night.

It is widely known that malaria burden is not homogenous across spaces, mainly due to variations in local environmental variables [12–14]. For example, the Tanzania malaria indicator report [15] indicates that malaria prevalence varies between regions, ranging from 0 to 24% [15]. It further postulates that most people now live in areas with less than 5% parasite prevalence, the greatest burden being in the north and southern areas, with the central belt being mostly low-prevalence. However, even within the high transmission districts, some households are at higher risk of malaria transmission than other households in the same villages [15]. Intervention strategies should be designed to capture these variations [16, 17]. Various studies have yielded evidence on environmental factors and malaria transmission [18–20], yet the information on the contribution of each factor to small scale variations of malaria transmission is still limited. However, this kind of heterogeneity may affect the efficacy of control strategies, specifically when transmission is low.

Malaria hotspots play a critical role in perpetuating transmission, even in regions with seasonal transmission patterns [21]. Consequently, focusing on these areas is anticipated to result in a substantial reduction in malaria transmission. Additionally, the mapping of these hotspots can facilitate the prediction of transmission patterns within villages, thereby enhancing the planning of interventions. Several studies investigating hotspot analysis propose that their occurrence is linked to environmental factors, such as proximity to mosquito breeding habitats. However, despite hotspots being observed at various levels, including the household level, the integration of Geographic Information Systems (GIS) in predicting hotspots is often disregarded in models employed for mapping spatial malaria patterns in communities. Furthermore, effective malaria-targeted interventions in pockets of transmission could rely on information related to small-scale variations. Therefore, it is imperative to identify and target those areas that sustain high malaria transmission, usually called hotspots [22]. The evidence suggests that targeting hotspots can lead to a decrease in malaria risk and transmission [21, 23]. Significant effort is required to understand the malaria transmission intensity at the local level and help identify and predict the local distribution of human populations at risk of malaria.

Regarding the causes and effects, it is known that more than 70% of malaria transmission risks are associated with environmental variables [21, 24, 25]. These variables include temperature, vegetation cover, slope, altitude, distance to water bodies, humidity, human population, land use, and land cover (LULC), which provide favorable conditions for breeding sites for disease vectors [25, 26] as well as the growth of parasites. Based on this evidence, the development of geospatial techniques have been applied by epidemiologists and scientists to collect, manage, manipulate, visualize, identify, characterize, and monitor environmental variables [27, 28] for understanding the trend of diseases, including malaria. Geographic information systems (GIS) and Remote Sensing (RS) have been applied for modeling and understanding the temporal and spatial variations of environmental variables, and their relationship to disease vectors [29, 30] and position measures that can be taken to reduce their transmission. Remote Sensing refers to the process of acquiring information about an object, area, or phenomenon without being in physical contact with it [31]. It involves the use of various sensors, such as cameras and satellite systems, to collect data from a distance. GIS, on the other hand, offers capabilities in helping to identify malaria risk areas [12, 32, 33]. They have been used to detect how the interventions are distributed, assess abundances and distributions of the malaria vectors and their associated environmental factors, determine the distribution of insecticide resistance incidence, and map malaria transmission risks in general [17, 34]. Environmental data are widely analyzed and presented using the GIS technology, thus, information on fine-scale environmental factors can inform decision-making, provide guidance for malaria interventions at both district, and ward levels in Tanzania and beyond.

To achieve the goal of controlling and total elimination of malaria in Tanzania, it is important to develop a faster, more cost-effective, and efficient systematic program for malaria risk area prediction at the local level. The integration of advanced geospatial technology (GIS and RS) into malaria control measures would help to determine and predict malaria risk areas in a cost-effective manner, efficiently, and in a short time. Malaria risk area prediction facilitates efficient planning, control and management of the malaria disease.

This study aims to use a geospatial based approach to predict areas prone to malaria occurrence in Kilombero Valley which will eventually enable efficient allocation of financial resources for malaria control and optimal distribution of appropriate interventions.

## Methods

### Study area

**Fig 1:**
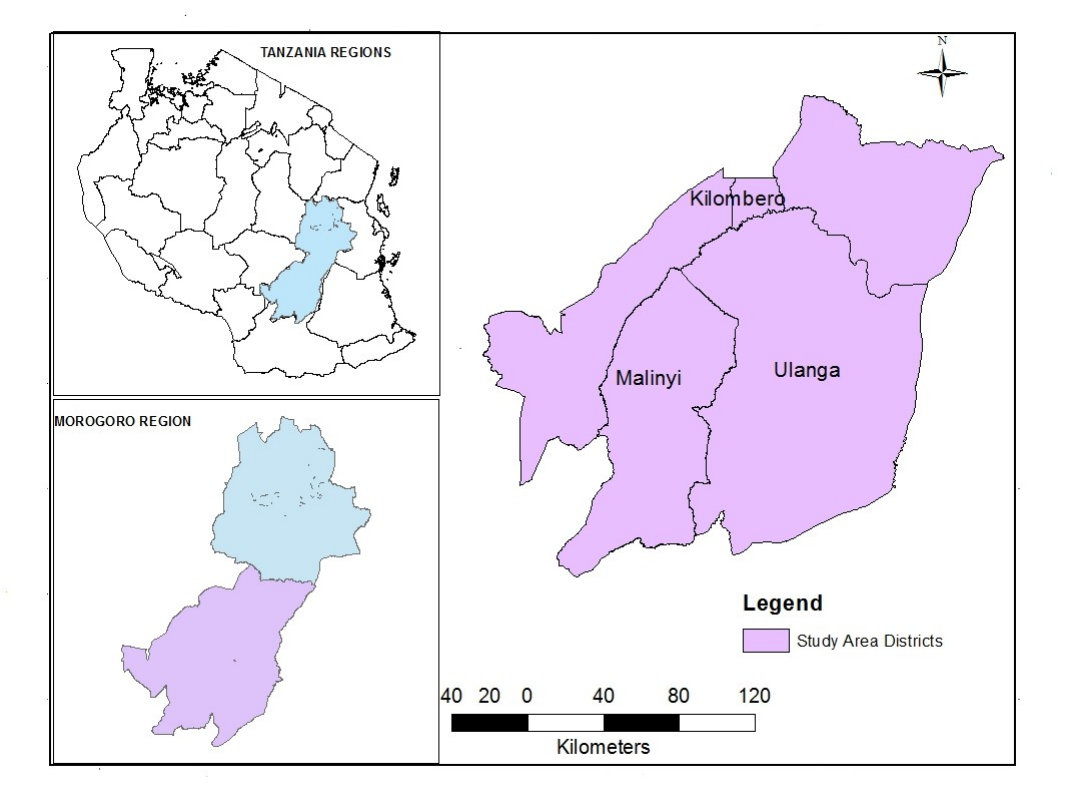
Map of the study area.

The study was conducted in three neighboring districts, specifically Kilombero, Ulanga and Malinyi (Kilombero Valley) which lies between latitude -7.6820 and -9.9990 to the north and south, respectively and longitude 35.3150 and 37.8120 to the west and east, respectively at 37S zone, South-eastern Tanzania (Fig 1). The area lies between the Udzungwa Mountains, which range to the northwest and Mahenge hills to the southeast and forms an intact natural wetland ecosystem comprising myriad rivers, which make up the lowland floodplain with an elevation range of approximately 109 m to 2636m above mean sea level.

The total coverage of the area is 35,436 km square which is equivalent to 54% of the total area of the Morogoro region and has a population of 673,083 people according to the 2012 national census [35]. Rainfall occurs year-round with two peaks; the “short rains” falling December to January, and the “long rains” in March to May with annual ranges between 1200 mm and 1800mm. Mean daily temperatures range between 20°C and 32°C, while relative humidity is between 70% and 90%. The main economic activities within the area are subsistence farming (rice and maize), keeping animals, and small-scale fishing in the Kilombero River and its tributaries [36].

The land has a high water table, which allows for the accumulation of standing water. This, in turn, easily facilitates the formation of mosquito breeding sites, leading to high densities of disease vectors. This area is a diverse and ecologically important region in Tanzania, characterized by its extensive wetlands and rich biodiversity. It encompasses a wide range of landscapes, including forests, grasslands, and floodplains, making it an ideal setting for studying various ecological and public health phenomena.

### Data acquisition and description

The study employed both primary and secondary data, which had an impact on the generation of the geospatial based model for malaria risk prediction in Kilombero valley (See Table 1). The data were collected from numerous sources based on the input required for the implementation of the predictive model. Data collected includes the locations of all public and private health centers that were downloaded free from the health portal of the United Republic of Tanzania, Ministry of Health, Community Development, Gender, Elderly, and Children, through the universal resource locator (URL) (http://moh.go.tz/hfrportal/). Human population data was collected from the 2012 population housing census (PHC) for the United Republic of Tanzania report.

**Table 1:** Variables associations with malaria risk and transmission.

Variable	Assumptions and Associations with Mosquitoes density, malaria risk and transmission from previous studies
Human Population Density and Distribution	There is a positive relationship between, human population, disease vectors and malaria risk. Activities performed by human being within settlement like cultivation and animal keeping both promotes the creation of breeding sites which favor the reproduction and growth of the vectors while animal keeping and human population ensure blood meal to some of the malaria mosquito species. Studies have shown a correlation between human population density and distribution and malaria incidence [18, 44–46].
Rainfall	The right amount of rainfall leads the presence of ground water as a potential breeding sites for mosquitoes and increasing humidity, which improves mosquito survival rates. Different *anopheles* mosquitoes prefer different types of water bodies in which to breed. In Tropical regions, water collections that support vector breeding appear mainly after the rainy season. Rainy season is a fertile period for the breeding sites, which are numerous. These species have the highest population density during the rainy season and these account for the high incidence of malaria at this period of the year. Several studies have established a positive association between rainfall, mosquito densities and malaria prevalence [13, 18, 47].
Altitude	Malaria transmission is strongly influenced by altitude. In general, malaria is more prevalent in low-lying areas and tends to decrease with increasing altitude. This is because the malaria parasite and its mosquito vector (*Anopheles* mosquitoes) have specific environmental requirements for breeding and survival. Higher elevations often have cooler temperatures, which are less favorable for mosquito development and the malaria parasite’s life cycle. Studies have consistently shown a negative relationship between altitude and malaria prevalence [14, 34, 48–50].
Land Use Land Cover (LULC)	LULC classes have influence on mosquito density and malaria transmission, mainly it affects the spatial and temporal distribution of mosquito larval habitant. Some LULC classes support habitat for mosquitoes and other not. LULC classes comprises of agriculture area, water bodies, forestry, settlement, bare land. Studies have associated between LULC classes and both malaria risk and transmission [51–56].
Curvature	Topographic curvature influences local microclimatic conditions such as temperature, humidity, and wind patterns. These factors can impact mosquito behavior, survival, and the development of the malaria parasite within the mosquito. For example, concave-shaped areas with less wind movement may have higher humidity and temperature, creating more favorable conditions for mosquito breeding and malaria transmission. Also topographic curvature can affect water accumulation and the formation of water bodies. Areas with positive topographic curvature (convex-shaped) tend to shed water more efficiently, resulting in less water accumulation and reduced mosquito breeding habitats. Conversely, negative topographic curvature (concave-shaped) areas can retain water, leading to the formation of stagnant pools and puddles that serve as suitable breeding sites for mosquitoes. Studies have shown the relationship between topographic curvature and malaria vectors [57, 58].
Slope	Topographic slope of the land affects the formation of stagnant water bodies, which are crucial breeding sites for mosquitoes. Flat or gently sloping terrains may create favorable conditions for the accumulation of rainwater, leading to the formation of suitable breeding habitats. Steep slopes, on the other hand, can limit the formation of such water bodies and consequently reduce mosquito breeding [58–60].
Aspect	Topographic aspect is the orientation that the slope faces, ranging from 0 to 360º. It determines the amount of sunlight that geographical area receives. The more sunlight receive can have a strong influence on temperature, which may affect mosquito larval survivorship and development. The association between aspect and malaria transmission may vary depending on local climate and ecological conditions [48, 57].
Proximity to the Stream Network	Streams network and other water bodies are predominant risk factor for malaria transmission because it can form vector-breeding sites for mosquitoes, increasing malaria transmission in their vicinity. Mosquitoes breed in the stagnant water pools formed by the stream network. Mosquitoes needs water to complete their aquatic life from egg, larva and pupa to adult emergence of both sexes. Therefore, stream network is an important factor influencing the occurrence and distribution of malaria cases in the areas [61–66].
Proximity to health Facilities	Malaria vulnerability in developing countries assumed to be less vulnerable to the area nearly to the health facilities canters due to easily access of the health services. Areas in close proximity to health facilities have simply and easy access to malaria diagnosis, treatment, and prevention measures. While the greater the distance from the health facility the higher prevalence of malaria [20, 67–69].
Proximity to the Road Network	There is a direct relationship between malaria prevalence and distance from road networks. The greater distance from the place to roads is estimated to be at highest risks to malaria while the place near to the roads having the lowest risk of malaria infections. The distance of a place from roads determines its accessibility to the social and health service as well as the effectiveness of intervention measures against malaria [20, 70].

Rainfall data were obtained from two local offices; Kilombero Agricultural Training and Research Institute (KATRIN) and Kilombero Valley Teak Company (KVTC). These offices collect meteorological data for agricultural purposes. Monthly data from 2012 to 2017 provided from thirteen (13) weather stations. Road and stream network shapefiles were downloaded free from the MapCruzin website via URL (https://mapcruzin.com/free-tanzania-arcgis-maps-shapefiles.htm).

With respect to the size of the study area, five neighboring scenes of the Landsat 8 OLI/TIRS images (path/row: 167/65, 167/66, 167/67, 168/66 and 168/67) were downloaded freely from the United States Geological Survey (USGS) website via URL: http://earthexplorer.usgs.gov. From July to November 2017 the images were selected and downloaded from the USGS Earth Explorer archive based on the lowest amount of cloud cover coverage as viewed from the archive before downloading. Finally, the digital elevation data with a spatial resolution of three arc-seconds (90m by 90m) using WGS 84 datum and the Geographic Coordinate System were downloaded free from the Shuttle Radar Topography Mission (SRTM) via URL (https://dds.cr.usgs.gov/srtm/version2_1/SRTM3/Africa/). Only six tiles that fall in the study area were downloaded, coded tiles as S08E035, S09E035, S10E035, S08E036, S09E036, S10E036, S08E037 S09E037 and S10E037. The analysis of this data complied with terms and conditions of the data sources. The classification of environmental variables and risk components like elevation, hazards, or malaria risk levels was done by assigning malaria risk categories to the environmental and demographic variables based on the current literature on the relationship between malaria transmission and environmental variables [37–43].

### Preparation and creation of model factor parameters

#### Creation of elevation factor

All six coded tiles were imported into the GIS environment for further analysis. Data management tools, with raster/raster data set/mosaic to new raster feature, was used to join the tiles and form an elevation map layer. Using the spatial analyst tool/reclassify feature, the generated elevation map was then classified into five classes as 109–358, 359–530, 531–747, 748–1017 and >1018 m.a.s.l. and new values were assigned for each class as 1, 2, 3, 4 and 5, respectively, with regards to the relationship with mosquito distribution and malaria risk. Finally, the elevation map based on malaria risk level is levelled as very high, high, moderate, low and very low respectively [37, 39–43].

#### Creation of slope factor

A slope map was created from the generated elevation map layer, using a spatial analysis tool/surface/slope feature. Also, the slope raster layer was further reclassified into five subgroups based on predefined slope classes using standard classification schemes, namely quantiles as 0–0.58, 0.59–2.90, 2.91–6.40, 6.41–14.54 and >14.54. This classification scheme divides the range of attribute values into equal-sized sub-ranges, which allow specifying the number of the intervals while the system determines where the breaks should be. The reclassified slope raster layer subgroups were ranked 1, 2, 3, 4 and 5 according to the degree of suitability for malaria incidence in the locality [58, 60]. To elaborate, the steeper slope values are related to lesser malaria hazards, and the gentler slopes are highly susceptible to malaria incidences [58, 60]. Finally, the slope map based on malaria risk level is leveled as very high, high, moderate, low and very low respectively [37, 39–43].

#### Creation of curvature factor

Curvature is another topographical factor that was created from the generated elevation map using the spatial analysis tool/surface/curvature feature. The curvature raster layer was further reclassified into five subgroups based on predefined curvature class. The reclassified curvature raster layer subgroups were ranked to 1, 2, 3, 4 and 5 according to their degree of suitability for malaria occurrence. To explain, this affects the acceleration and deceleration of flow across the surface. A negative value indicates that the surface is upwardly convex, and flow will be decelerated, which is related to being highly susceptible to malaria incidences. A positive profile indicates that the surface is upwardly concave and the flow will be accelerated which is related to a lesser malaria hazard, while a value of zero indicates that the surface is linear and related to a moderate malaria hazard [58]. Lastly, the curvature map based on malaria risk level is leveled as very high, high, moderate, low, and very low respectively [37, 39–43].

#### Creation of aspect factor

As a topographic factor associated with mosquito larval habitat formation, aspect determines the amount of sunlight an area receives [48, 57]. The more sunlight received the stronger the influence on temperature, which may affect mosquito larval survival. The aspect of the study area also was generated from the elevation map using spatial analyst tools/ raster /surface /aspect feature. The aspect raster layer was further reclassified into five subgroups based on predefined aspect class. The reclassified aspect raster layer subgroups were ranked as 1, 2, 3, 4 and 5 according to the degree of suitability for malaria incidence, and new values were re-assigned in order of malaria hazard rating. Finally, the aspect map based on malaria risk level is leveled as very high, high, moderate, low, and very low, respectively [37, 39–43].

#### Creation of human population distribution factor

Human population data was used to generate a population distribution map related to malaria occurrence. Kilombero valley has a total of 42 wards, the data was organized in Ms excel 2016 and imported into the GIS environment for the analysis, Inverse Distance Weighted (IDW) interpolation in the spatial analyst tool was applied to interpolate the population distribution map. The population distribution map was further reclassified into five subgroups based on potentiality to malaria risk. The reclassified map layer subgroups were ranked according to the vulnerability to malaria incidence over a locality such as areas having high population has the highest vulnerability [18, 44] and the less population has less vulnerable, and the new value was assigned as 1, 2, 3, 4 and 5. Then leveled as very high, high, moderate, low and very low malaria risk level, respectively [37, 39–43].

#### Creation of proximity to health facilities factor

The distribution of health facilities has a significant impact on the malaria vulnerability of the population dwellings in the Kilombero valley [20, 67–69]. The health facility layer was created by computing distance analysis using proximity multiple ring buffer features in spatial analyst tool/multiple ring buffer. Then the map layer was reclassified into five sub-layers such as within (0–5) km, (5.1–10) km, (10.1–20) km, (20.1–50) km and >50km. Later on, the new values were assigned as 1, 2, 3, 4 and 5. Then reclassified as very high, high, moderate, low and very low malaria risk levels, respectively [37, 39–43].

#### Creation of proximity to road network factor

The distance to the road network is also a significant factor, as it can be used as an estimation of the access to present healthcare facilities in the area [20, 70]. Buffer zones were calculated on the path of the road to determine the effect of the road on malaria prevalence. The road shapefile of the study area was inputted into GIS environment and spatial analyst tools / multiple ring buffer feature were used to generate five buffer zones with the interval of (0–5) km, (5.1–10) km, (10.1–20) km, (20.1–50) km and >50km regards to the effect of road on malaria occurrence. New values were assigned as 1, 2, 3, 4 and 5, and reclassified as very high, high, moderate, low and very low malaria risk level, respectively [37, 39–43].

#### Creation of distance from stream network factor

Streams provide water to flood plains and stagnant water that increases the breeding sites for vector diseases that lead to malaria transmission and risk at a locality [18, 62, 64, 66]. Proximity to stream map was created using GIS environment system, spatial analyst tools with the application of multiple ring buffer feature, the feature was set to generate the output layer at different distance intervals such as (0–1) km, (1.1–3) km, (3.1–5) km, (5.1–10) km and >10km. The reclassification was done based on mosquito flight range [71] and new values were assigned as 1, 2, 3, 4 and 5. Then reclassified as very high, high, moderate, low and very low malaria risk level, respectively [37, 39–43].

#### Creation of rainfall distribution factor

Rainfall leads to the presence of groundwater as a potential for breeding sites, which increases mosquito and malaria transmission [13, 18, 47, 72]. The data provided in the excel sheet was analyzed by finding the average of the rainfall quantity of about 5 years ago. The data was imported into the GIS environment and IDW spatial analyst interpolation tool was applied to interpolate the distribution of rainfall quantity received within the study area to generate a rainfall distribution map. The rainfall distribution map was further reclassified into five subgroups based on the predefined quantity of rainfall received. The reclassified map layer subgroups were ranked accordingly to the degree of suitability for malaria incidence in the locality. Example areas that receive more quantity of rainfall are related to higher malaria hazards and the less quantity of rainfall have less susceptible to malaria incidences, and the new value was assigned as 1, 2, 3, 4 and 5. Then reclassified values were leveled as very high, high, moderate, low and very low malaria risk levels, respectively [37, 39–43].

#### Creation of Land Use Land Cover (LULC) classes factor

LULC class map layer was generated from Landsat 8 images while undertaking this study. To fit the world reference system of the specific area, Erdas imagine 2014 (Hexagon Geospatial, USA) was used to geo-reference and project the satellite image to the WGS84 UTM zone 37S because the Landsat image is always north projected. Seven bands (Band 1, 2, 3, 4, 5, 6 and 7) in all five scenes were stacked differently using the raster-spectral layer/stack feature into a single multispectral image which contains a higher degree of spectral resolution and five scenes were then mosaicked using mosaicpro feature. Raster subset image feature was applied in which the study area shapefile and mosaicked image were manipulated and produced an image that fit the study area extent. Raster supervised classification feature was used to perform a supervised classification method for classifying the image into basic classes such as water bodies (stream network), forestry, grassland and mixed feature. The mixed feature class is a class in which both settlements, cultivated land, bushes, bare land and scrubs were merged as one class feature. The features were combined due to the low resolution of the Landsat image that makes these features look the same during the classification process. The classified image was exported into the GIS environment for further ranking and reclassification. Also, the LULC class map was reclassified in four subgroups for their susceptibility and Suitability for malaria risk.

#### Generation of the predicted areas prone to malaria occurrence

Generation of the geospatial based model to predict areas prone to malaria occurrence was done on the basis of risk computation model by Shook *et al*. [73], that state risk is the product of both hazard, vulnerability and element at risk as presented in the Eq 1.

R=H×E×V (1)

Where:

R = Risk, E = Element at risk and V = Vulnerability, H = Hazard

Malaria vulnerability analysis was performed in model builder by weight overlay of two map layers such as proximity to health facilities map and proximity to road network map, with 0.6 and 0.4 weight respectively. The output was reclassified into five sub-layers and a new value was assigned as 1, 2, 3, 4 and 5, then the reclassified map was leveled as very high, high, moderate, low and very low malaria vulnerability. Malaria hazard employed rainfall distribution map, proximity to stream map, elevation map, slope map, curvature map and aspect map as the factors of malaria incidence (Table 2). All factors were evaluated, weighted and ranked according to what seemed appraisable value for their importance to malaria hazard and Multiple Criteria Decision Making (MCDM) was employed using free Analytical Hierarchical Process (AHP) online software (web based system) with URL (http://www.isc.senshu-u.ac.jp/~thc0456/EAHP/AHPweb.html) to calculate the weights and consistency index (CI). The computed Eigen vector, which is an output of the pairwise comparison matrix to produce a best fit set of weight. The weight module presented in the Table 2 while the consistency ratio (CR) of the calculated Eigen vector was 0.099087, since it is less than 0.10 hence the judgment is acceptable. The consistency index (CI) measures the extent of inconsistency in the judgments provided by a decision-maker during the pairwise comparison process. It is calculated based on the principle that the relative importance of criteria or alternatives should remain consistent throughout the decision-making process. The computed Eigen vector was used as a coefficient for the respective factor layers to be combined in weighted overlay analysis in the GIS environment. After assigning weight according to their importance for each parameter (Table 2), the hazard layer was computed by overlaying the six selected hazard parameter factors in GIS environment. Then the hazard layer was advanced reclassified into five subclasses as: 1, 2, 3, 4, 5, and the reclassified sub groups were ranked as: very high, high, moderate, low and very low respectively. According to the ranking in this study a rank 1 was used to show the parameter was very hazardous or risky, and a rank 5 was used as to show the parameter was less hazardous. The element at risk layer was generated by overlaying two layers namely LULC map and human population distribution map, with 0.4 and 0.6 weight respectively on the basis of malaria susceptibility to all elements. Moreover, the element at risk layer was reclassified into five subclasses as: 1, 2, 3, 4, 5 and the reclassified map was leveled as: very high, high, moderate, low and very low respectively [37, 39–43].

**Table 2:** Showing malaria hazard weighted ranking.

S/N	Factor	Weight	Value (Sub factor)	Ranking	Hazard
1	Rainfall	0.462563	2.80–3.05	5	Very Low
			3.06–3.21	4	Low
			3.22–3.45	3	Moderate
			3.46–3.84	2	High
			3.84–4.44	1	Very High
2	Elevation	0.139481	109–358	1	Very High
			359–530	2	High
			531–747	3	Moderate
			748–1017	4	Low
			>1017	5	Very Low
3	Slope	0.0735481	0–0.58	1	Very High
			0.59–2.90	2	High
			2.91–6.40	3	Moderate
			6.41–14.54	4	Low
			> 14.54	5	Very Low
4	Distance to Stream	0.260906	0–1	1	Very High
			1.1–3	2	High
			3.1–5	3	Moderate
			5.1–10	4	Low
			>10	5	Very Low
5	Curvature	0.0390291	-2.35 –(-0.86)	5	Very Low
			-0.876 –(-0.014)	4	Low
			0.015–0.021	3	Moderate
			0.022–0.093	2	High
			0.0934–2.232	1	Very High
6	Aspect	0.0244668	–1–71.17	1	Very High
			71.18–143.34	2	High
			143.35–215.52	3	Moderate
			215.53–287.69	4	Low
			287.70–359.87	5	Very Low

Using MCDA while having only a few criteria (variables), the decision-making process can become overly simplified and may not capture the complexity of the real-world situation. This can lead to biased or incomplete evaluations. MCDA is particularly useful when having a range of factors to consider, as it helps account for various aspects of the decision problem. When there are only a few criteria, the importance of each one might be self-evident, and using MCDA might be unnecessary. Therefore, in this study, MCDA was only applied in the assigned weight for the variables used to generate the malaria hazard map while in the other variables and components used to generate both, the vulnerability map, the element at risk and malaria risk map the weights were assigned through a combination of expert knowledge and literature reviews to avoid the bias or incomplete evaluation of both variables and components.

**Fig 2:**
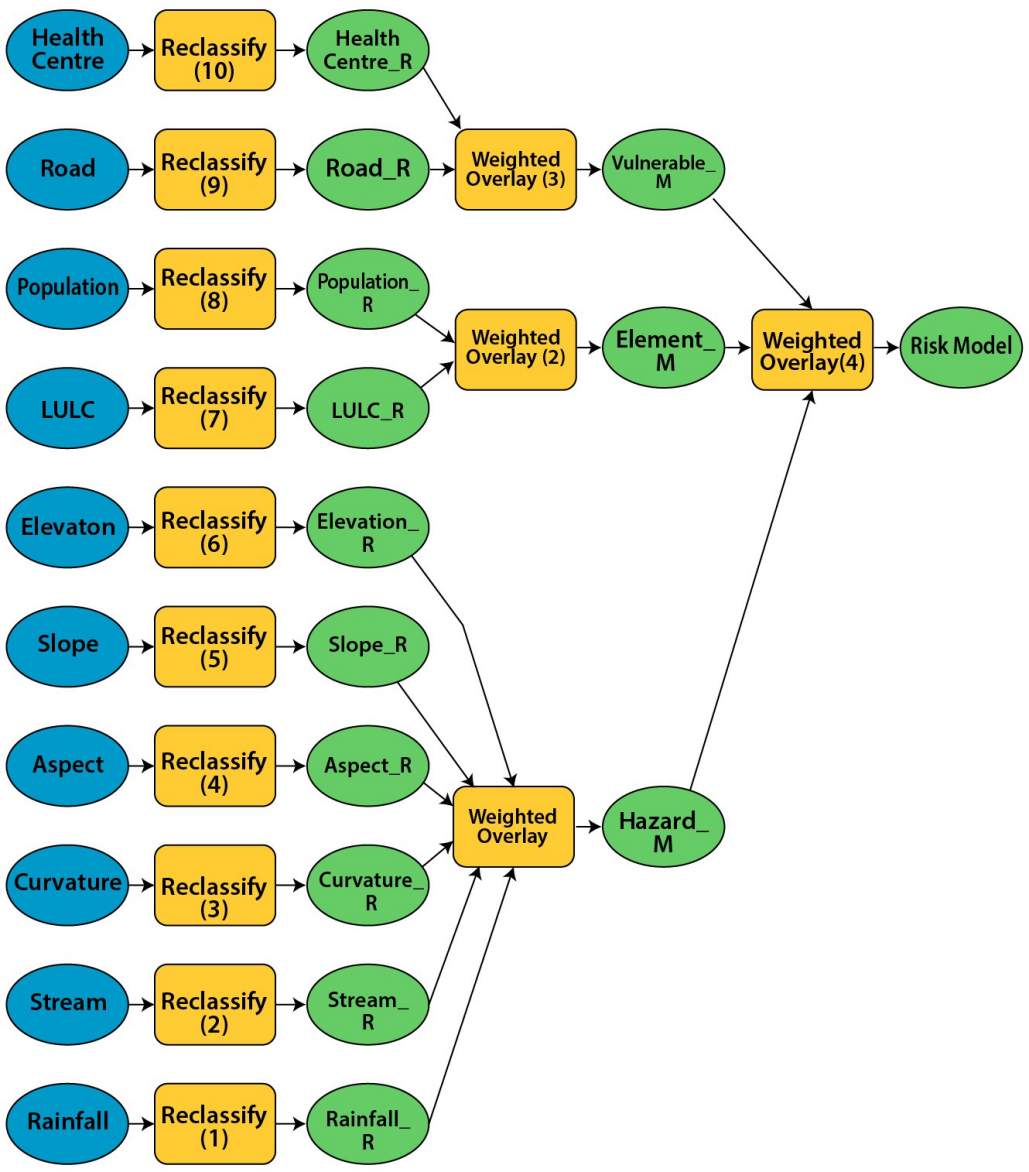
Systematic representation of malaria risk analysis in model builder tool.

Furthermore, all three components of risk were taken with weight of 0.4, 0.3 and 0.3 for element at risk, hazard and vulnerability factor respectively with respect to malaria occurrence at locality. Then weighted overlay analysis was performed in model builder for the generation of predicted areas prone to malaria and it was reclassified according to the risk level into three sub groups as high, moderate and low malaria risk areas. Fig 2 demonstrates the process of generating predicted areas prone to malaria using a model builder tool in the GIS environment [37, 39–43].

#### Ethical considerations

Ethical approval was provided by Institutional Review Board (IRB) of Ifakara Health Institute approval number IHI/IRB/NO: 06–2016; and the Medical Research Coordinating Committee of the National Institute for Medical Research, in Tanzania with approval number NIMR/HQ/R.8a/VOL1X/2218. The permission to publish this study was granted by the director general of National Institute of Medical Research in Tanzania (Ref: NIMR/HQ/P.12 VOL XXXV/118).

## Results

### Malaria hazard areas

**Fig 3:**
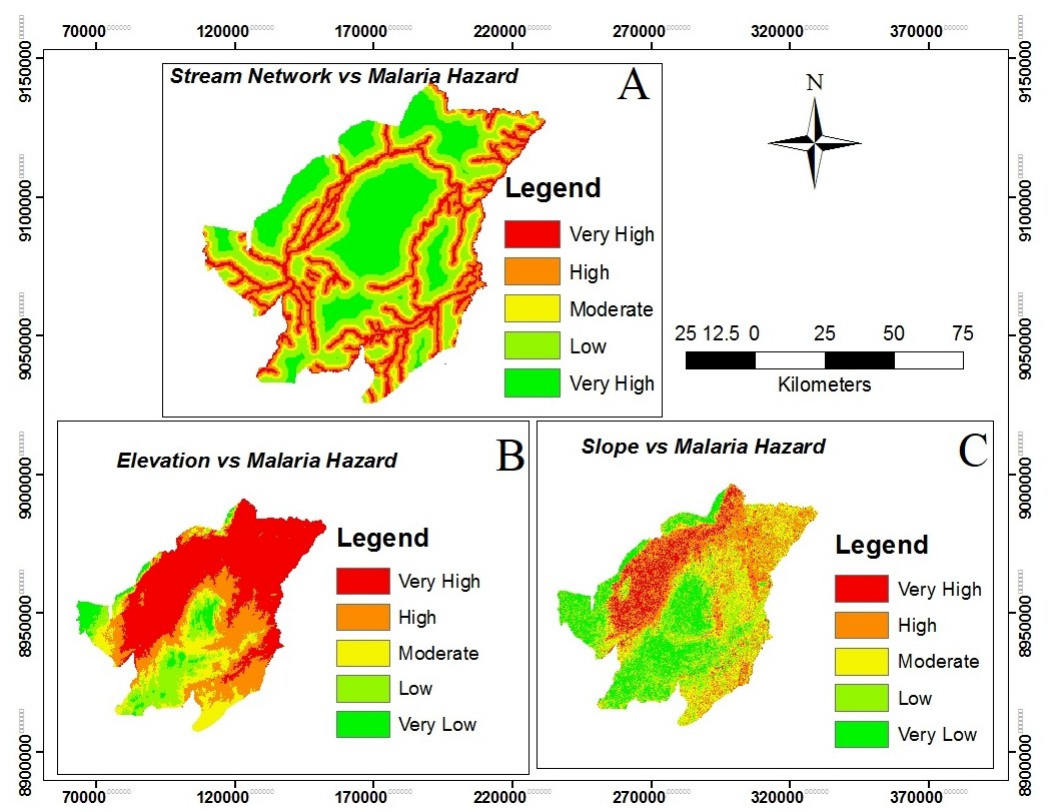
Illustrating the relationship between key environmental variables and the malaria hazard levels. The maps depict the following factors: examines the relationship between the proximity to the stream network and malaria hazard levels (Panel A), the correlation between elevation and malaria hazard levels (Panel B) and (Panel C) explores the relationship between slope and malaria hazard levels.

**Fig 4:**
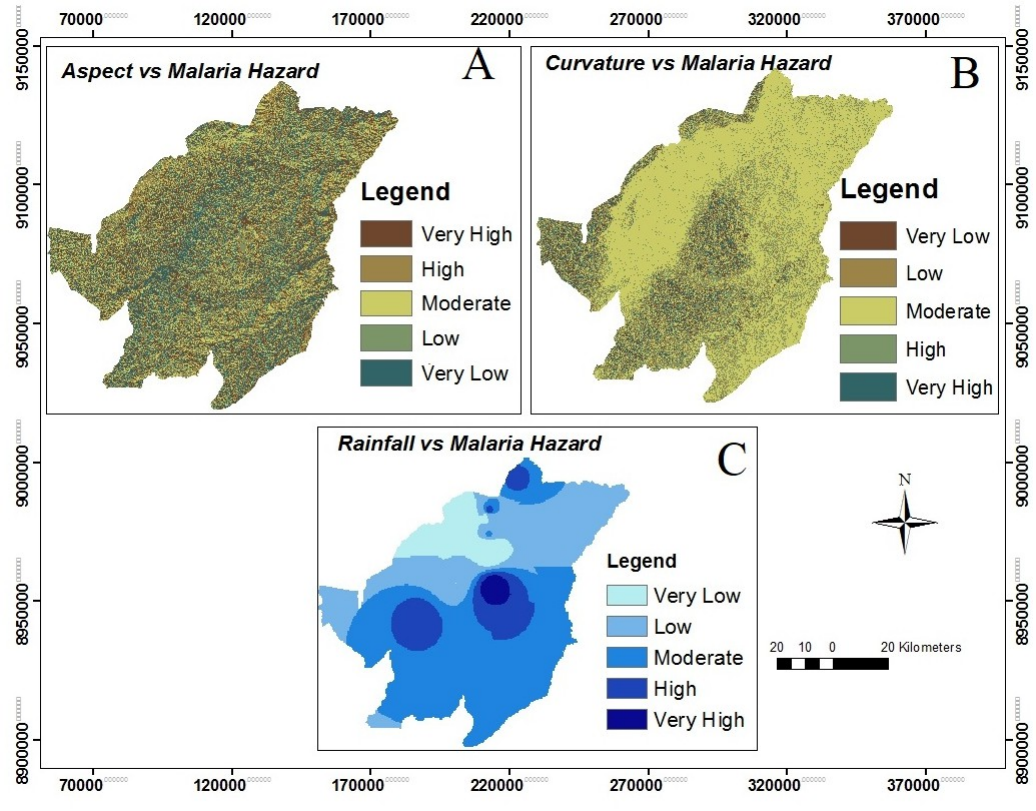
Illustration of the relationship between specific environmental variables and malaria hazard levels. The maps depict the following factors: the correlation between aspect and malaria hazard levels (Panel A), the association between curvature and malaria hazard levels (Panel B), and the relationship between rainfall and malaria hazard levels (Panel C).

The study used variables such as elevation map, slope map, aspect map, curvature map, rainfall map and proximity to stream network map to be the predictors for malaria hazard prediction in Kilombero valley (Figs 3 and 4 with all panels). These determinants have a significant contribution to malaria hazard levels in the study area. The maps provide information that malaria hazard is higher in an environment with lower elevation (higher temperature), an abundance of rainy lands, gentle slopes and availability of stagnant waters around streams.

Malaria hazard map show that 51%, 40% were subjected to moderate and high malaria hazard areas respectively while less than 10% of the area fall in very high, low and very low malaria hazard areas (Fig 6, panel A). Also the results demonstrate that much of the study area falls within moderate and high malaria hazard level, therefore it is implying that *Anopheles* mosquitoes can survive in almost every part of the study area as a result of malaria risk and transmission respectively.

### Malaria vulnerability

Malaria vulnerability refers to the susceptibility of individuals, populations, or geographic regions to the transmission and impact of malaria. Proximity to health facilities and road networks are among the factors that contribute to malaria vulnerability in the locality [74, 75].

**Fig 5:**
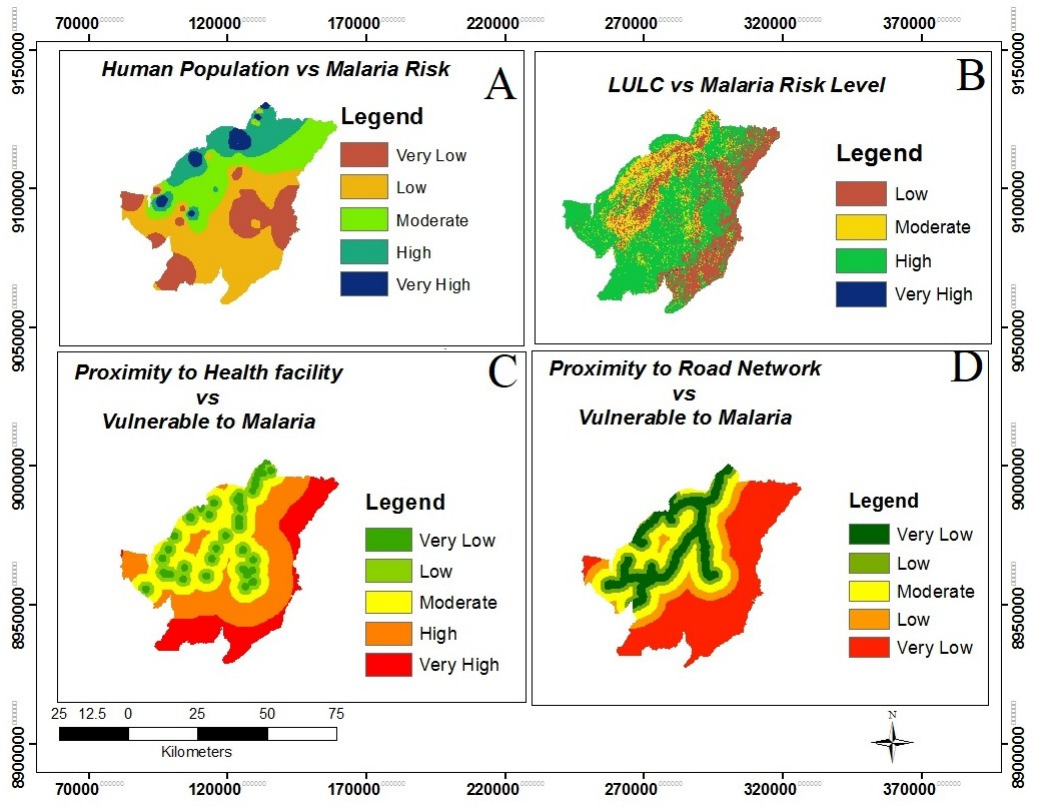
Illustration of the relationship between human population distributions and the vulnerability levels to malaria (Panel A), the correlation between different land use and land cover classes and the malaria vulnerability levels (Panel B), the relationship between the proximity of health facilities and the malaria vulnerability levels (Panel C) and the association between the proximity of road networks and malaria vulnerability levels (Panel D).

Based on the health facility proximity map, it demonstrates that, about 70% of the study area fell in moderate, high and very high vulnerable to malaria while only 30% being in low and very low vulnerable to malaria incidence (Fig 5, panel C and Table 3). Also proximity to road network displays that about 50% of the area falls in the high and very high malaria risk, in view of the fact that people living far from road network are facing difficult to access and driver of social and health services while only 26% of the area is within low and very low malaria incidence due to easy access to social and health services (Table 3 and Fig 5, panel D).

**Table 3:** Summary of the results shows the level of malaria vulnerability factors.

S/N	Parameter	Very High	High	Moderate	Low	Very Low
1	Proximity to Health Facilities	18%	30%	22%	18%	12%
		6417 km	10467 km	7659 km	6557 km	4350 km
2	Proximity to Road Network	42.4%	10.5%	18.6%	12.9%	15.2%
		15008.6 Km	3723.2 km	6592.1 km	4540.4 km	5398.9 km

**Fig 6:**
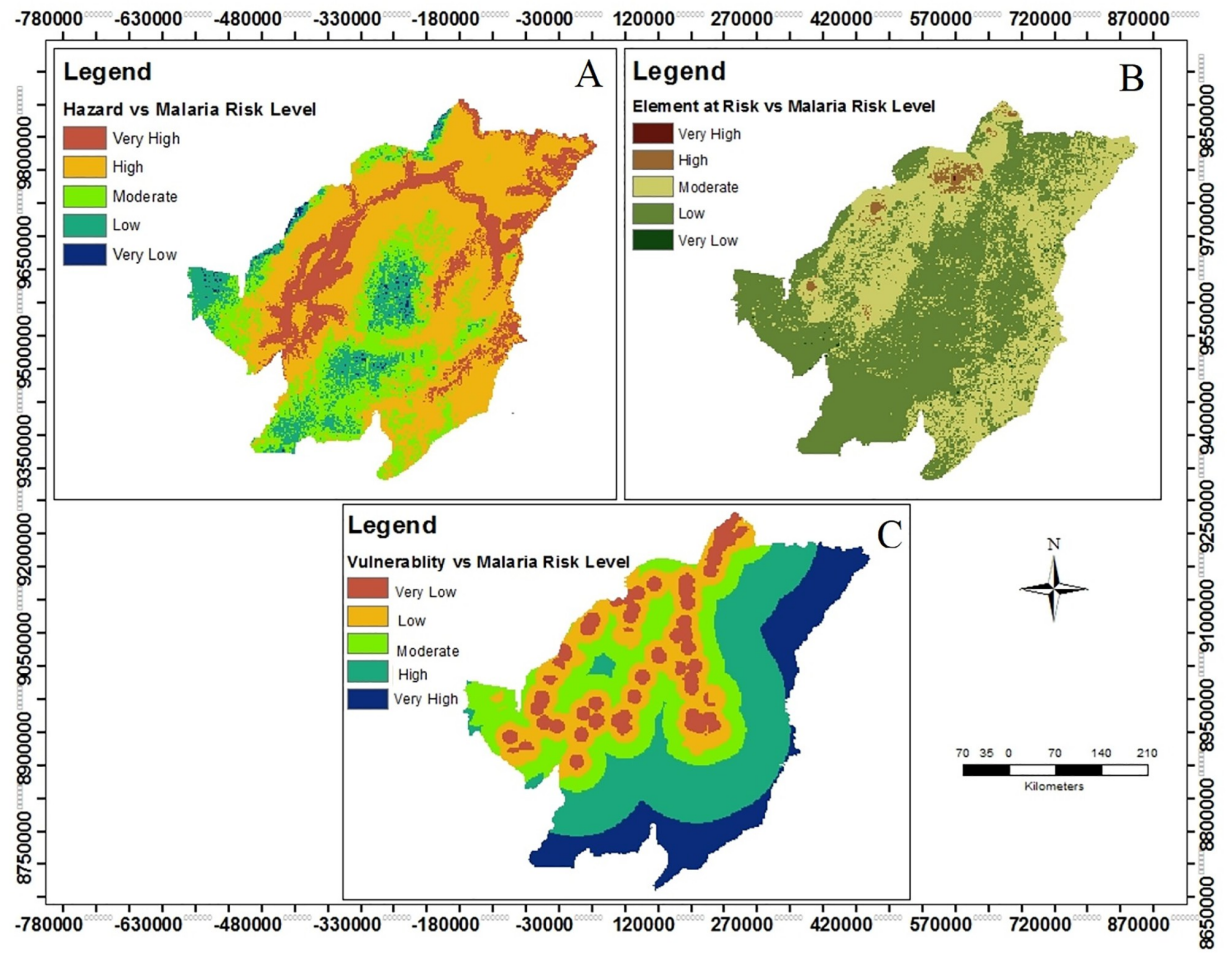
Illustration of the relationship between malaria risk components and malaria risk levels, the correlation between hazard component and malaria risk levels (Panel A), the relationship between element at risk and malaria risk levels (Panel B) and the association between malaria vulnerability and malaria risk levels (Panel C).

Also the product of both distance to health facilities and distance to road network layers as malaria vulnerability at study area shows that 31% of the area is in high vulnerable for malaria, very high malaria vulnerable area covers around 18%, 21% counts for moderate vulnerable to malaria while low and very low malaria accounts for 20% and 10%, respectively (Fig 6, panel C), The findings demonstrate that about 70% of the area is in moderate, high and very high vulnerable to malaria and only less than 30% of the area is covered with low and very vulnerable for malaria.

### Elements at risk

Elements at risk is a generic term that signifies everything that might be exposed to hazards phenomena in a particular area, either directly or indirectly. Ranging from buildings to the economy and from individual persons to the community’s environment. The study focused on human population distribution and LULC class layers as the primary components for elements at risk.

Based on the findings, the LULC class layer demonstrate that mixed feature covered by 28% of the study area while grassland class accounted with 17.2% coverage, forestry class cover the largest area of all LULC classes with 54.4% and water bodies being the last with less than one percent coverage (Fig 6, panel B). With regards to vulnerability to malaria, the mixed feature class was labeled as very high risk areas due to their suitability for both malaria vector and parasite, water bodies were considered as high risk areas while grassland and forestry were labeled as moderate and low malaria risk areas respectively.

Human population distribution map indicates that about the entire area is in low and very low malaria risk level. 49.4% and 45.2% are in low and very low malaria risk levels respectively while only 6% of the area is covered by moderate, high and very high malaria risk levels (Fig 5, panel A). Likewise, the findings demonstrate that the southern part of the area is covered with very low population distribution compared to the northern part which is covered with low population and some small parts are moderate, high and very high, therefore malaria incidence is high in the northern part of the study area compared to the southern part.

According to the combination of both LULC classes and human population maps as element at risk map, the findings demonstrate that elements is exposed to malaria occurrence at 0.02%, 1.65%, 40.88%, 57.31% and 0.15% respectively, therefore most part of the area is predominance to low and moderate malaria risk level compared to high, very high and very low malaria incidence (Fig 6, panel B).

### Areas prone to malaria occurrence

**Fig 7:**
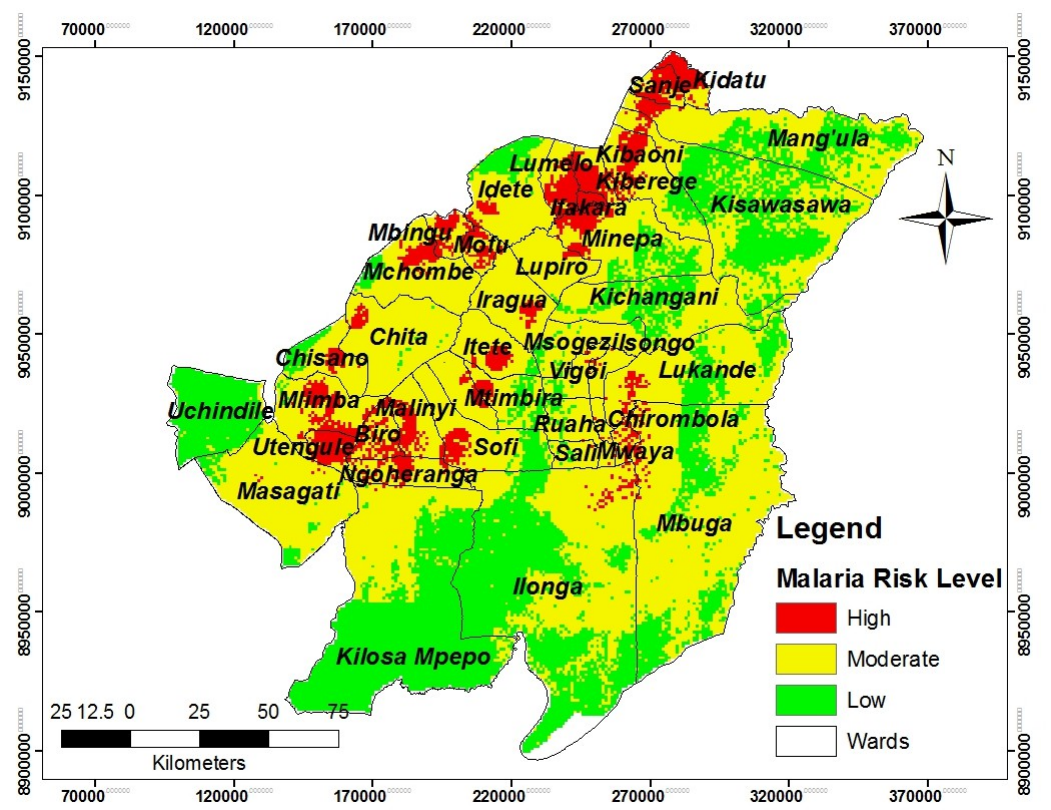
Malaria risk map showing the status of wards in the study area.

Based on the results, malaria risk map show that 8%, 61% and 31% of the study area were subjected to high, moderate and low malaria risk areas respectively (Fig 7). This shows that the majority of study areas fell in moderate risk level (61%) and followed by low risk level (31%) while high malaria risk area covers a small area, which occupies only 8% of the total area (Fig 7 and Table 4).

**Table 4:** Indicates malaria risk rating, area coverage and percentage.

S/N	Rating	Area (m^2^)	Area (km^2^)	Percent (%)
1	High	2631946145	2632	8
2	Moderate	21195530000	21196	61
3	Low	10867700000	10868	31

## Discussion

The risk of malaria transmission is strongly associated with environmental variables [26, 72, 76], which influence the growth and development of both mosquito and parasite respectively. Geospatial technique has capability of collecting, integrating and understanding the temporal and spatial variations of the environmental variables and its relationship to disease vectors and position measures that can be taken to reduce its transmission [13].

Previous studies confirmed a negative relationship between altitude and mosquito abundance in a certain area. Low elevation areas are associated with increased abundance of mosquito and high transmission of malaria disease compared to the high elevation areas [49]. The altitude of Kilombero valley determines the amount of rainfall water receives, and increases the water bodies like streams in study area which form suitable breeding sites for disease transmitting mosquitoes. Furthermore, it is known that climatic factors, like temperature, change over an increasing elevation and thus the most favorable conditions for both mosquito vectors and parasites [19, 24, 57]. The range of 20°C–30°C is the temperature range for the survival and growth of both malaria vector and parasite while below 16°C or above 40°C can cause death [77]. Temperature decreases with 0.6 degrees Celsius per 100m rise [57], therefore at some altitudes it can get too cool for successful mosquito reproduction.

Areas with gentle slope or flat are always having the variant number of stagnant or low movement of water that can influence the presence of breeding sites and increase the abundance of disease vectors [58–60]. This leads to high risk and transmission of malaria. Steeper slope areas allow faster movement of water and affects the stability of water which does not favor the presence of the breeding sites and decrease the number of disease vectors as a result of low malaria transmission [58]. This study shows that about 80% of the area falls on gentle slopes and such a situation may accelerate chances for water stagnation which may encourage breeding and survival of mosquitoes which leads to high risks of malaria transmission.

Stream network are an example of water a body, which has already been revealed to be a predominant risk factor for malaria transmission because it can form standing and stagnant water that provides sites for mosquito breeding. Mosquitoes always require water to complete the aquatic stage of their lifecycles, from egg, larva and pupa to adult [19]. Therefore, proximity to streams is an important factor influencing the occurrence and distribution of malaria cases in the locality. People who live near the stream network are at risk of being bitten by mosquitoes compared to people who live far from the stream network. With regards to this study, shows that people who live near the Kilombero river and its attributes are at higher risk of getting malaria disease compared to people who live far from the rivers.

Kilombero valley is indeed one of the regions in Tanzania that experiences a significant range of rainfall throughout the year. Rainfall leads to the presence of groundwater as a potential breeding site for *Anopheles* mosquitoes which increases the abundance of mosquito and malaria occurrence in study area [18, 47, 63]. This study shows that from the center downward part of the study area received more rainfall compared to the upward which implies that the southern parts of the study area are at higher risk of malaria compared to the other part of the area.

It’s known that topographical aspects in the locality can have a strong influence on temperature [57]. This is because of the angle of the sun in the northern and southern hemispheres which is less than 90 degrees or directly overhead which determines the amount of sunlight an area receives. The more sunlight received can have a strong influence on temperature, which may affect mosquito larval survivorship [48, 57]. With regards to the topographical aspect of the study area, it shows that most of the area receives the same amount of sunlight which implies that the effect of topographic aspect to malaria risk is the same to all risk levels.

Study area comprises about 100 private and government health facilities including dispensaries, health centers and central hospitals. Health facilities distribution has a significant impact on the malaria incidence of the population dwellings in the locality [20, 67–69]. This study shows that, some of the areas there is a negative association between human population distribution and distance from health facilities which may result in malaria risk and incidence. According to WHO reports and other existing studies show that the distance to health facilities plays a significant role in determining the vulnerability of malaria occurrence. When populations live within closer proximity to health stations (within 3 km), they are considered less vulnerable to malaria [67, 69]. This is attributed to easier access to health services, which can lead to prompt diagnosis and treatment, mosquito control measures, and health education. On the other hand, areas located beyond the 3 km radius of health facilities are considered more vulnerable to malaria. The lack of convenient access to healthcare resources can lead to delayed diagnosis and treatment, allowing malaria to spread more easily and become more prevalent.

Physical cover on the earth’s surface and the arrangement, activities and inputs people undertake in a certain land cover type to produce change often had effects on malaria vectors [78]. Agricultural land like mature maize fields, rice fields and pastured grasslands are positively related to the presence of larval habitats and results in malaria risk and transmission at the locality, while nonagricultural land like shrubs and bare land are negatively associated with the presence of larval habitats [51, 56, 78, 79].

Human activities such as cultivation and animal keeping have an impact on both malaria risk and transmission intensity. Crop cultivations create breeding sites that favor the reproduction and growth of mosquitoes [13, 18, 47] while animal keeping and human population provide blood meal to some of the mosquito species [80]. Therefore, in areas with a high existence of human population malaria incidence can be high, compared to area where few or no people live [81]. The current study shows that there is a positive correlation between human population distribution and malaria risk in some parts of the area, for example the Ifakara ward is the area with a high number of human population distribution and the map depict the area is at higher malaria risk.

The final malaria risk map shows that all parameters used in this study had different weight influences for the malaria occurrence in the study area. Nevertheless, the human population distribution was the most dominant factor for the prevalence of malaria since most of the area was not covered by human population. The fact that most of the area was not covered by human population highlights the significance of this factor. In areas where there are fewer or no humans, there might be fewer opportunities for the malaria parasite to be transmitted, as the disease is primarily spread through the bite of infected mosquitoes.

Based on this study, shows that only Ifakara ward which is located in the northern part of the study area, falls under the high malaria risk level. Also, the study revealed that Utengule, Mlimba, Kidatu, Kibaoni, and Lumemo wards exhibit a dual risk level, falling into both high and moderate-risk categories. Moreover, the model categorizes the wards like Uchindile, Kichangani and Msogazi falls in both moderate and low malaria risk levels as well as the model depict that Masagati, Itete and Kisawasawa wards fall in all three malaria risk level. Furthermore, the model demonstrates that some of the areas were covered by small or isolated hotspots which were observed in Lukande, Chilombora, Mbunga, Ilonga and Biro wards (Fig 7).

While the model incorporates various environmental factors, it does not directly measure or establish the association between these factors and malaria transmission cases. We highlight the need for further research, including field studies and data collection on malaria transmission cases, to strengthen the evidence and establish the direct link between the identified variables and malaria transmission hazard. It is important to note that our study did not incorporate actual malaria or mosquito data specific to the study area. Consequently, the interpretability of the results in terms of malaria risk and mosquito distribution should be considered with caution. Incorporating such data would have enhanced the accuracy and reliability of our findings. Also, the mixed feature class comprises diverse land use and land cover (LULC) classes, and its inclusion in our analysis may introduce some complexity and potential limitations. The inclusion of the mixed feature class in our analysis is a reflection of the complex nature of the study area and the need to account for the heterogeneity of LULC patterns.

Generated areas prone to malaria occurrence revealed that the study areas were subjected to all three risk levels such as high, moderate and low malaria risk areas respectively. Also, the model shows that more than 50% of the study area is at risk of malaria disease in the fact that 8% and 61% of the study area is within high and moderate risk of malaria respectively. Therefore, under current environmental variables that are associated with malaria transmission, the findings provide the basis for assessing the progress of control and show which geographic areas should be prioritized.

## Conclusion

This study demonstrates that most of the area is hazardous to malaria transmission because a significant portion of the study area falls within moderate and high malaria hazard levels. The factors for the malaria risk map coincide with the factors for mosquito growth which implies that *Anopheles* mosquitoes can survive in almost every part of the study area hence high risk of malaria transmission.

Furthermore, this study has shown the importance and usefulness of different tools like weighted overlay tools in the GIS environment as Multi Criteria Evaluation techniques for indicating areas at different ratings in terms of both malaria hazard and malaria risk respectively. Additionally, the AHP online system which was applied for factor weight computation by providing a series of pairwise comparisons of the relative importance of factors to the suitability of pixels for the malaria risk prediction, has generated valuable information. This could be useful for disaster control and decision-making in the future. Therefore, the study confirms the approach used was capable of integrating all the malaria hazard causative factors and the components of malaria risk as well in a GIS environment.

The study highlights the importance of geospatial techniques for predicting malaria transmission risks by relying on analyzing various environmental factors using RS and GIS. These techniques were used to create predicted areas prone to malaria transmission based on the environmental parameters, reclassification, overlay and identification of risk levels (High, moderate or low). Therefore, geospatial techniques could be utilized to facilitate timely and effective vector control and prevention programs.

## List of abbreviations

AHP: Analytical Hierarchical Process
GIS: Geographic Information System
IDW: Inverse Distance Weighted
IRS: Indoor Residual Sprays
KATRIN: Kilombero Agricultural Training and Research Institute
KVTC: Kilombero Valley Teak Company
LLINs: Long-Lasting Insecticide-Treated Bed Nets
LULC: Land Use and Land Cover
MCDM: Multiple Criteria Decision Making
PHC: Population Housing Census
RS: Remote Sensing
SNP: School Net Program
SRTM: Shuttle Radar Topography Mission
TMIS: Tanzania Malaria Indicator Survey
TNVS: Tanzania National Voucher Scheme
UCC: Universal Coverage Campaigns
URL: Universal Resource Locator
USGS: United State Geological Survey
WHO: World Health Organization
